# Evaluation of Trail-Cameras for Analyzing the Diet of Nesting Raptors Using the Northern Goshawk as a Model

**DOI:** 10.1371/journal.pone.0127585

**Published:** 2015-05-20

**Authors:** Gonzalo García-Salgado, Salvador Rebollo, Lorenzo Pérez-Camacho, Sara Martínez-Hesterkamp, Alberto Navarro, José-Manuel Fernández-Pereira

**Affiliations:** 1 Ecology and Forest Restoration Group, Department of Life Sciences, University of Alcalá, Sciences Building, Alcalá de Henares, Madrid, Spain; 2 Independent Field Biologist, Villa de Caldueño (Llanes), Asturias, Spain; 3 Independent Field Biologist, Castrelo*-*Cela (Bueu), Pontevedra, Spain; University of Lleida, SPAIN

## Abstract

Diet studies present numerous methodological challenges. We evaluated the usefulness of commercially available trail-cameras for analyzing the diet of Northern Goshawks (*Accipiter gentilis*) as a model for nesting raptors during the period 2007–2011. We compared diet estimates obtained by direct camera monitoring of 80 nests with four indirect analyses of prey remains collected from the nests and surroundings (pellets, bones, feather-and-hair remains, and feather-hair-and-bone remains combined). In addition, we evaluated the performance of the trail-cameras and whether camera monitoring affected Goshawk behavior. The sensitivity of each diet-analysis method depended on prey size and taxonomic group, with no method providing unbiased estimates for all prey sizes and types. The cameras registered the greatest number of prey items and were probably the least biased method for estimating diet composition. Nevertheless this direct method yielded the largest proportion of prey unidentified to species level, and it underestimated small prey. Our trail-camera system was able to operate without maintenance for longer periods than what has been reported in previous studies with other types of cameras. Initially Goshawks showed distrust toward the cameras but they usually became habituated to its presence within 1–2 days. The habituation period was shorter for breeding pairs that had previous experience with cameras. Using trail-cameras to monitor prey provisioning to nests is an effective tool for studying the diet of nesting raptors. However, the technique is limited by technical failures and difficulties in identifying certain prey types. Our study also shows that cameras can alter adult Goshawk behavior, an aspect that must be controlled to minimize potential negative impacts.

## Introduction

Knowledge of the diet of a species is essential to understanding its biology and establishing appropriate conservation and management strategies [1], but diet assessment is methodologically challenging [2]. Conventional diet assessment of raptors involves locating, collecting, preserving and identifying unconsumed or undigested remains of prey [3]. Several factors make these indirect methods prone to serious biases when assessing diet.

First, behavioral and physiological characteristics of the raptor species in question can strongly influence the types of prey remains available for analysis. For example, species swallowing their prey whole leave fewer food remains but more signs in pellets [3]. Diurnal raptors usually leave pellets containing fewer bones than do nocturnal raptors, due to their lower stomach pH [4, 5]. Second, the type of prey involved also strongly influences the types of remains available for analysis. Raptors usually pluck birds thoroughly, often beginning this process at the place of capture, which can lie far from the nest; mammals, in contrast, are delivered to the nests less skinned [6–8]. Some tender or digestible prey, such as amphibians, reptiles, and juveniles in general, give rise to fewer uneaten remains and fewer signs in pellets [9, 10]. Third, environmental conditions can significantly affect the prey remains available for analysis. Pellets and other remains are less detectable in humid and rainy environments than in dry ones [11] and they decompose faster, with the decay rate varying according to size and consistency [3]. Thus, when applied to diurnal raptors, analysis of food remains such as feathers, fur, bones and scales, tends to overestimate the contribution of large prey species and birds and therefore underestimate the contribution of small prey and of mammals, reptiles and amphibians [12–14]. All these biases argue for the use of direct methods that can record prey items when they are delivered to the nest by the adults.

Advances in camera technology and the growing availability of affordable recording devices has led to increases in the use of cameras placed near the nest, replacing human observers hidden in blinds [15–17]. The use of cameras can reduce costs, improve prey identification (because observations are made at much closer range), allow a larger sample of nests to be monitored for longer periods, and create a visual archive that can be reviewed later for different research objectives [18–20]. The use of cameras can yield more accurate estimates of prey provisioning rates, prey biomass and degree of prey consumption than indirect methods can [21]. However, using cameras in diet studies also has several drawbacks. Image capture and analysis systems require specialized knowledge and do not work equally well for all species, field conditions and research questions [22]. In fact, the identification rate of prey to species level from camera/video images is often lower than from analysis of prey remains, especially for small prey [16, 23]. Raptor diet is often studied during the breeding season, when bird activities focus on the nest and so it is easier to survey prey items [3]; as a result, the risk of research disturbance is high when installing cameras. The literature on camera-based research disturbance is limited, and most published studies have examined relatively few nests for short periods [24]. In addition, relatively few studies have examined the causes and frequencies of camera failures in the field [22, 25, 26]. These identification, disturbance, and technical concerns highlight the need to develop reliable camera systems that can operate stably for long periods, avoiding the need for additional visits to the nest site after installation and thereby minimizing research disturbance during the breeding season.

Here we describe a long-term, large-scale study to evaluate the usefulness of commercially available trail-cameras for assessing the diet of breeding Northern Goshawks (*Accipiter gentilis*, Linnaeus, 1758; hereafter Goshawk) as a raptor model. Cameras were installed in 80 Goshawk nests and used to monitor prey deliveries. At the same time, we performed indirect analyses of four types of prey remains collected from nests and plucking sites: pellets, bones, feather-and-hair remains, and feather-hair-and-bone remains combined. Our goals were three-fold. First, we compared the ability of all 5 methods to identify prey to species level and to discriminate individuals within each species in order to assess diet composition. Such comparative analysis of direct and indirect methods is important for understanding biases in diet estimation (*e*.*g*., [8, 21, 27, 28]). Second, we analyzed the performance of the trail—cameras, evaluating their operating times and the frequencies and types of technical failure. Third, we explored the impact of camera surveillance on the behavior of adult Goshawks. We focused on adults because nestlings did not appear to pay any attention to the cameras in our study.

## Materials and Methods

### Area and species under study

The study was carried out in a 400 km^2^ densely populated coastal area (480 inhabitants / km^2^) in the NW of Spain (Morrazo peninsula and Terra de Cotobade, Galician region, 42°20´N, 8°47´E). The climate is humid—oceanic, with annual average precipitation of 1586 mm and an annual average temperature of 14.4°C [29]. Wooded areas occupy 51% of the territory and are dominated by exotic forest plantations (*Eucalyptus globulus*).

The Goshawk is a medium—sized, diurnal, forest—dwelling raptor that shows strong territorial behavior [30]. It is distributed extensively throughout the Holartic region, where it preys upon a wide variety of medium—sized birds and mammals. Breeding goshawks usually have several nests within their territory, which can be occupied alternately in different years. During the breeding season this species use plucking sites located near the active nests. The density of the breeding population of Goshawks is high in the study area (10.5 active territories / year / 100 km^2^). Breeding pairs nest considerably high on *Eucalyptus* trees (average nest height, 22.4 m, range 8–35 m, *n* = 64) [31, 32]. Nest stand tree density (stem diameter at breast height > 15 cm) averages 420 ± 16 trees/ha. Percentage bush cover in the understorey is 65%, being fern, blackberry, ivy, and gorse the most common species.

All the work was conducted in accordance with relevant national and international guidelines, and conforms to the legal requirements of the regional government (Dirección Xeral de Conservación da Natureza of the Xunta de Galicia) which granted permission to carry out the study. Special efforts were made to minimize disturbance to animals (see *Data collection* section below). Research disturbance was investigated as part of the aims of the present study (see *Results* and *Discussion* sections below).

### Data collection

From 2007 to 2011 we visited all active Goshawk nests in the study area (42 nesting territories) during the second half of the breeding period to collect all uneaten prey remains and pellets available. Nest platforms were checked twice each year, once in May-June, when nestlings were banded and trail-cameras installed (mean nestling age 23.7 ± 3.4 SD days; by this age nestlings are able to thermoregulate and feed by themselves), and again after fledging (September), when cameras were retrieved (young Goshawks fledge at about 42 days old, [30]). Plucking sites and nest surroundings were thoroughly checked 3 times (May-June, July-August, and September).

We pooled all prey remains collected in the three visits. The pool of prey remains was divided into 3 subsamples (feather-and-hair, bones, and pellets), which were analyzed separately. We identified feathers, hairs and bones to species level and estimated the minimum number of individuals of each prey species, adjusting for repetition of principal feathers and bones. The degree of feather development was also used to differentiate individuals since it allows distinguishing among nestlings, fledglings and adults. We also estimated the diet from feather-hair-and-bones combined, since this method is the most widely used, together with pellet analysis, for studying raptor diet [33]. In this case, to avoid multiple counting of individuals, we used only the type of remains in which we detected the highest number of individuals for the given prey species. The proportions of bird and mammal remains within each pellet (*n* = 677) were determined. Mammals in pellets were identified to the level of species or group of species, but birds were not because we failed to develop a reliable method for identifying small feathers and fragments of feathers from the pellets that did not overestimate the most easily identifiable species [11, 34].

We installed one digital trail—camera in 80 nests where prey remains had been collected. We performed a pilot study with 3 cameras in 2007 (Bushnell Trailscout 2.1Mp, 1 GB memory card), and then we installed 18–21 cameras each year from 2008 to 2011 (Moultrie Game Spy I40, 4 GB memory card). Cameras were programmed to take one picture per minute after being triggered by motion of the nestlings or the adults in the nest; otherwise, the cameras did not take pictures. In 2007 and 2008, cameras with the factory colors of dark brown or black were used; in subsequent years, cameras were painted in camouflage colors before installation. No components of the photographic system were installed on the ground. Details about the cameras and their installation are given in S1 Appendix. While camera installation itself took fewer than 45 min, difficulties in reaching the nests meant that we were usually present at nest sites for nearly 3 hours. Nestlings were removed from the nest during camera installation and manually fed with chicken or rabbit to minimize potential impacts due to the temporary absence of adults. For the same reason, we also left extra food in the nests after we had finished installation and returned the nestlings.

Since we were unable to identify every prey to species level from camera images, we classified them into three taxonomic classes (bird, mammal, reptile) and 14 taxonomic subcategories. Unidentified prey, which we defined as a group of prey that could not be identified even to class level, were classified into size classes: very small (<100 g), small (100–184 g), medium (185–400 g) and large (>400 g).

### Statistical analysis

Prey data over the study period were summed for each of the 5 analysis methods and each nesting territory. From the 42 nesting territories sampled we selected 20 territories where we were able to form a complete picture of diet, based on at least 15 pellets (mean ± SD, 33.9 ± 20.2 pellets per territory) and 20 prey items from each one of the other four type of remains (41.2 ± 15.8 prey per territory from feather-and-hair remains; 31.7 ± 13.5 from bones; 57.5 ± 19.0 from feather-hair-and-bones; and 133.5 ± 45.9 from cameras). We used repeated—measures ANOVA to compare diet estimates: the percentage of individuals of each prey category served as the response variable, prey analysis method as the fixed factor and territory as the repeated factor. Proportion data were arcsine-transformed prior to statistical comparison. Where sphericity could not be assumed based on the results of Mauchly's sphericity test, the degrees of freedom were adjusted using the Greenhouse—Geisser correction. Repeated—measures analysis allowed us to separate the effect of method from the effect of territory. Bonferroni—adjusted pairwise comparisons were performed for each factor that was found to be significant in the ANOVA at *p* < 0.05.

To evaluate camera performance we measured the number of days of operation, number of images captured, and the type and frequency of technical problems affecting different device components. To evaluate the effect of the cameras on adult behavior, we adopted two approaches. One was to estimate the number of days required for each breeding pair to become habituated to the camera (days-to-habituation). The breeding adults in our study exhibited some distrust towards the cameras, which led to both low frequency visits and prey provisioning to the nest immediately after camera installation. These frequencies increased progressively afterwards, until they approximately stabilized at a characteristic value for each breeding pair. We considered that adults had become habituated to the camera the first day that this value was reached. Thus, we defined days-to-habituation as the number of days needed for the daily number of visits to the nest and numbers of prey provided to stabilize after camera installation. Another approach was to determine how long passed between when the camera was installed in a nest and when the adults entered for the first time (hours-to-entry for the first visit to the nest) and how long passed between when the camera was installed in a nest and when they provided the first prey (hours-to-entry to provide the first prey). We compared these hours-to-entry with how long it took adults to do these activities on a typical day after habituation to the camera. To estimate the hours-to-entry on a typical day after habituation, we defined the time of day at camera installation as the reference time. For example, if the camera was installed at 18:00, we measured how long it took to observe these activities after that time on the typical day after habituation to the camera. The comparisons were made using the Wilcoxon matched—pairs test.

We analyzed whether painting the cameras with camouflage colors reduced the days-to-habituation and hours-to-entry. These comparisons were made using the Mann—Whitney U test. Since non-camouflaged cameras were used during the first years of the study (2007–2008) and camouflaged cameras later on (2009–2011), we used data only from birds which had had no previous experience with our cameras in order to exclude the effect of experience. This group comprised 37 adult females, which we identified by reading their field—readable rings in camera images. Further details of adult Goshawk trapping and marking in the study area can be found in [32].

We also analyzed whether previous experience with cameras affected adult behavior in subsequent years. We used the Kruskal—Wallis test to compare the average days-to-habituation and hours-to-entry for breeding females of known identity in the first year (no previous experience, *n* = 38), second year (some experience, *n* = 12) and subsequent years (significant experience, *n* = 10).

## Results

### Comparison of diet estimates obtained from direct and indirect methods

Of the five methods that we used to analyze Goshawk diet, camera images allowed us to detect 2664 prey items, compared to 824 items detected in feather-and-hair remains, 633 in bones, and 1150 in feather-hair-and-bone pools. This implies that a large proportion of prey items known to be delivered to nests (based on camera images) was not detected in analyses of prey remains; this proportion was at least 69% in analysis of feather-and-hair remains, 76% in analysis of bones, and 57% in analysis of feather-hair-and-bone combined. The proportion of unidentified prey in samples of feathers-and-hair and of bones was <1% of all prey (Table 1), while the corresponding proportion from camera images was nearly 20% (513 of 2664). Smaller prey were the most difficult to identify from camera images: 49% of unidentified prey were very small (<100 g), 37% were small (100–184 g), 14% were medium (185–400 g), and none was large (>400 g).

**Table 1:** Proportion of prey in the Goshawk diet and comparison of prey analysis methods.

		Weight (g)	*df*	*F*	*p*	Pellets	Feather-hair remains	Bone remains	Feather-hair-bone remains	Camera images
Birds	**Small passerines**	<50	1.16	18.53	**<0.001**	-	3.0 ± 0.8^**a**^	0.0^**b**^	2.1 ± 0.5^**c**^	0.2 ± 0.1^**b**^
	**Exotic birds (psittacines)**	<100	1.18	7.20	**0.011**	-	0.8 ± 0.3	0.0	0.6 ± 0.2	0.1 ± 0.1
	**Common Blackbird, Spotless Starling, *Turdus spp*., Great Spotted Woodpecker**	65–85	1.94	45.51	**<0.001**	-	14.1 ± 1.9^**a**^	1.6 ± 0.7^**b**^	10.4 ± 1.5^**c**^	8.6 ± 0.8^**ac**^
	**Eurasian Magpie**	180	1.96	6.58	**0.004**	-	10.0 ± 1.8^**a**^	6.4 ± 1.5^**b**^	8.5 ± 1.5^**ab**^	5.5 ± 1.0^**b**^
	**Eurasian Jay, European Green Woodpecker**	175	2.15	30.72	**<0.001**	-	27.6 ± 3.2^**a**^	10.1 ± 2.0^**b**^	21.0 ± 2.4^**c**^	23.8 ± 1.3^**ac**^
	Eurasian Sparrowhawk	150–300	1.85	0.14	0.853	-	0.3 ± 0.3	0.3 ± 0.2	0.3 ± 0.2	0.3 ± 0.2
	**Pigeons and doves**	185–400	3.00	178.71	**<0.001**	-	33.1 ± 3.2^**a**^	72.7 ± 2.8^**b**^	47.0 ± 2.6^**c**^	30.6 ± 1.7^**a**^
	Poultry	>400	2.02	1.08	0.351	-	0.7 ± 0.3	0.4 ± 0.3	0.7 ± 0.2	0.5 ± 0.2
	**Carrion Crow**	>400	2.25	3.34	**0.040**	-	0.7 ± 0.3	1.1 ± 0.5	0.9 ± 0.3	0.1 ± 0.1
	Yellow-legged Gull	>400	1.79	0.52	0.581	-	1.7 ± 0.9	2.1 ± 1.4	1.8 ± 1.0	0.9 ± 0.5
Mammals	Rats and micromammals	20–150	*NT*	*NT*	*NT*	2.6 ± 0.2	0.1 ± 0.1	0.3 ± 0.1	0.3 ± 0.1	0.4 ± 0.1
	**Red Squirrel**	250	2.89	15.34	**<0.001**	4.8 ± 0.8^**ac**^	2.1 ± 0.4^**bc**^	1.5 ± 0.5^**b**^	2.2 ± 0.4^**bc**^	7.4 ± 1.4^**a**^
	European Rabbit	250–1000	2.23	1.84	0.167	0.9 ± 0.4	1.2 ± 0.3	1.5 ± 0.5	1.6 ± 0.3	1.5 ± 0.3
Reptiles	Ocellated lizard		*NT*	*NT*	*NT*	0.0	0.0	0.0	0.0	0.1 ± 0.0
**Unidentified prey**			1.22	292.89	**<0.001**	-	0.0^**a**^	0.1 ± 0.1^**a**^	0.1 ± 0.1^**a**^	19.4 ± 1.8^**b**^

Average proportion (% ± SEM) of prey in the Goshawk diet estimated from analyses of pellets, prey remains (feather-and-hair, bone, and feather-hair-and-bone), and camera images. Prey categories were defined based on taxonomic groups and mean prey weight. Unidentified prey could not be classified even to class level as bird, mammal or reptile. Prey analysis methods were compared using repeated-measures ANOVA (n = 20 nesting territories) and Bonferroni-adjusted pairwise comparisons (differences between groups are indicated by different superscript letters). Significant differences (*p* < 0.05) are shown in bold. *NT* = Not tested

Birds were the most abundant prey, accounting for 88 to 97% of the identified prey items depending on the method; mammals, in contrast, accounted for 3 to 12% of prey items (Table 2). Analysis of camera images and pellets estimated lower percentages of birds and higher percentages of mammals than the methods based on uneaten prey remains (feather-and-hairs, bones, feather-hair-and-bones combined). Cameras were the only analysis method that detected reptiles, which were detected at quite low levels and were always identified to be the Ocellated Lizard *Timon lepidus*.

**Table 2:** Proportion of birds, mammals and reptiles in the Goshawk diet and comparison of prey analysis methods.

	*df*	*F*	*p*	Pellets	Feather-hair remains	Bone remains	Feather-hair-bone remains	Camera images
**Birds**	2.62	22.02	**<0.001**	91.3 ± 1.4^**a**^	96.5 ± 0.5^**b**^	96.6 ± 0.7^**b**^	95.9 ± 0.6^**b**^	87.8 ± 1.5^**a**^
**Mammals**	2.61	21.79	**<0.001**	8.7 ± 1.4^**a**^	3.5 ± 0.5^**b**^	3.4 ± 0.7^**b**^	4.1 ± 0.6^**b**^	12.1 ± 1.5^**a**^
Reptiles	*NT*	*NT*	*NT*	0.0	0.0	0.0	0.0	0.1 ± 0.0

Average proportion (% ± SEM) of birds, mammals and reptiles in the Goshawk diet estimated from analyses of pellets, prey remains (feather-and-hair, bone, and feather-hair-and-bone), and camera images. Unidentified preys were excluded from these calculations. Prey analysis methods were compared using repeated-measures ANOVA and Bonferroni-adjusted pairwise comparisons (see Table 1 legend for details).

The ability of each method to detect prey varied with species size. The proportion of very small (50–99 g) and small (100–184 g) prey was highest in feather remains, lowest in bones, and intermediate in camera images and feather-hair-and-bone remains (Table 3). Furthermore, the bone remains and the cameras detected nearly no extremely small prey (<50 g). Medium-sized prey (185–400 g) were particularly abundant in bone remains (73% of them), mainly pigeons and doves (*Columba livia f*. *domestica*, *C*. *palumbus*, *Streptopelia decaocto*, *S*. *turtur*). All 5 prey analysis methods yielded similar estimates for large prey (> 400 g). The same relationships between methods and prey species size were observed when only bird prey were analyzed.

**Table 3:** Proportion of prey in the Goshawk diet according to five size classes and comparison of prey analysis methods.

Weight of prey (g)	*df*	*F*	*p*	Feather-hair remains	Bone remains	Feather-hair-bone remains	Camera images
**< 50**	1.43	9.70	**0.002**	3.2 ± 0.9^**a**^	0.3 ± 0.2^**b**^	2.4 ± 0.6^**ac**^	0.6 ± 0.2^**bc**^
**50–99**	1.92	46.47	**<0.001**	14.9 ± 2.0^**a**^	1.6 ± 0.7^**b**^	11.0 ± 1.6^**ac**^	8.7 ± 0.8^**c**^
**100–184**	2.00	31.10	**<0.001**	37.7 ± 3.8^**a**^	16.5 ± 2.5^**b**^	29.6 ± 3.0^**c**^	29.3 ± 1.7^**c**^
**185–400**	2.14	111.89	**<0.001**	35.6 ± 3.5^**a**^	74.6 ± 2.7^**b**^	49.6 ± 2.8^**c**^	38.3 ± 1.9^**a**^
> 400	2.06	1.06	0.36	4.4 ± 1.1	5.1 ± 1.6	5.0 ± 1.2	3.0 ± 0.7

Average proportion (% ± SEM) of prey in the Goshawk diet according to five size classes estimated from analyses of pellets, prey remains (feather-and-hair, bone, and feather-hair-and-bone), and camera images. Unidentified preys were excluded from these calculations. Prey analysis methods were compared using repeated-measures ANOVA and Bonferroni-adjusted pairwise comparisons (see Table 1 legend for details).

The ability of each method to detect mammals also varied with the size of species (Table 1). The smallest mammals (rats and micromammals, 20–150 g) were detected almost exclusively in pellets, whereas the cameras recorded larger quantities of larger mammals such as squirrels (*Sciurus vulgaris*). This probably reflects the fact that raptors require a longer handling time to process these larger preys, which increases the number of images of each prey improving its identification. Thus, the proportion of squirrels ranged from 1.5 to 7.5% and varied with prey analysis method as follows: uneaten prey remains (bones, hairs, bones-and-hairs) < pellets < camera images. All 5 methods gave similar estimates for rabbits.

### Trail-camera performance

Although 80 cameras were installed in the study area, 2 were stolen, so we could include data from only 78 in our analyses. In our 2007 pilot study, the median number of images per camera was 1948 and the interquartile range (*IQR*) of 25^th^-75^th^ percentiles was 1899–1953, whereas in 2008–2011 we obtained a median of 6458 and *IQR* of 4700–7554 images (S1 Appendix, S3 Dataset). The images occupied an average of 97% of the memory card in 2007 and 61% of the card in subsequent years.

Nearly half of the cameras (44%) functioned correctly from their installation until the nestlings fledged, operating a median (*IQR*) of 59 (23–89) days. This long operating time is related to the fact that many of these cameras kept functioning long after fledging. When the nestlings fledged, the number of camera images decreased drastically and prey deliveries to the nest occurred only sporadically. Thus, the median time during which these cameras were actually registering prey deliveries to the nest was 19 (13–26) days. The remaining 56% of the cameras experienced some kind of malfunction before fledging, operating for a median of 10 (8–13) days, and registering prey deliveries for a median of 9.5 (8–12.5) days. Operating time depended mainly on the type of external battery and memory card capacity: the time was longer when new lead—acid batteries and 4 GB cards were used in 2009 and 2011. Among the cameras with these features (n = 38), the percentage that functioned well until fledging increased to 68%. These cameras that functioned well until fledging had a median operating time of 60.5 (31–90) days. Table 4 shows the most frequent types of technical failures of the trail—cameras and their incidence.

**Table 4:** Types of technical failure of the trail—cameras (n = 78).

Failure description	Incidence*
Energy depletion	42.3
Interruptions of image capture lasting a few hours presumably due to technical failure rather than a lack of activity in the nest. This failure usually occurred toward the end of the camera operating life, when battery level was low	33.3
Existence of some low-quality images (too dark or too bright)	16.7
Technical failures of unknown origin	15.4
Obstacles in the field of view or images providing only partial views of the nest due to difficulties during camera installation	12.8
Failure of camera bracket, leading to misframing. This mainly affected unreinforced camera brackets used before 2009. Overall failure rate was 20% for unreinforced brackets and 5% for reinforced brackets	9.0
Memory card full	9.0
Built-in limit of 9999 pictures was reached, preventing further image recording	8.0
Camera installed too distant from the nest, leading to less frequent motion triggering and therefore to an incomplete dataset	6.4
Failure of infrared flash	5.1
Entry of water into the camera, fogging the lens for a certain period of time	5.1
Camera installed too close to the nest, leading to slightly blurred pictures and more frequent motion triggering, ultimately causing premature depletion of the battery or memory card	2.6
Obstructions of the field of view due to branch and leaf growth	2.6
Failure of the male-plug used to connect external batteries (in 2008 only)	NA

* Percentage of cameras affected. Sometimes the same camera suffered several problems simultaneously.

### Impact of trail-camera monitoring on nesting Goshawks

The installation of trail-cameras caused no nest desertions by breeding Goshawks during the study period. The hours-to-entry interval (median, *IQR*) between our leaving the nest site after camera installation and the first activity of adults detected in the nest was 4.8 (2.2–13.9) hours and between our leaving the nest and the first recorded delivery of prey was 14.4 (4.6–23.2) hours; these intervals varied substantially among pairs. Once the adults had become habituated to the cameras, the estimated hours-to-entry intervals were significantly shorter: 0.8 (0.2–2.4) hours for the first activity and 3.1 (1.2–9.3) hours for the first recorded delivery of prey (Wilcoxon T = 344 and 466, respectively, with *p* < 0.001 in both cases). More than 75% of breeding pairs started to conduct these activities within 24 hours after we left the nest site, although 2 pairs (2.6%) took approximately 48 h to enter the nest the first time after camera installation. Neither painting the cameras with camouflage colors nor previous experience with cameras reduced the time for the first activity of adults in the nest or for the first delivery of prey (Mann—Whitney U test, *p* = 0.90 and 0.50; Kruskal—Wallis test, *p* = 0.75 and 0.76).

**Fig 1:**
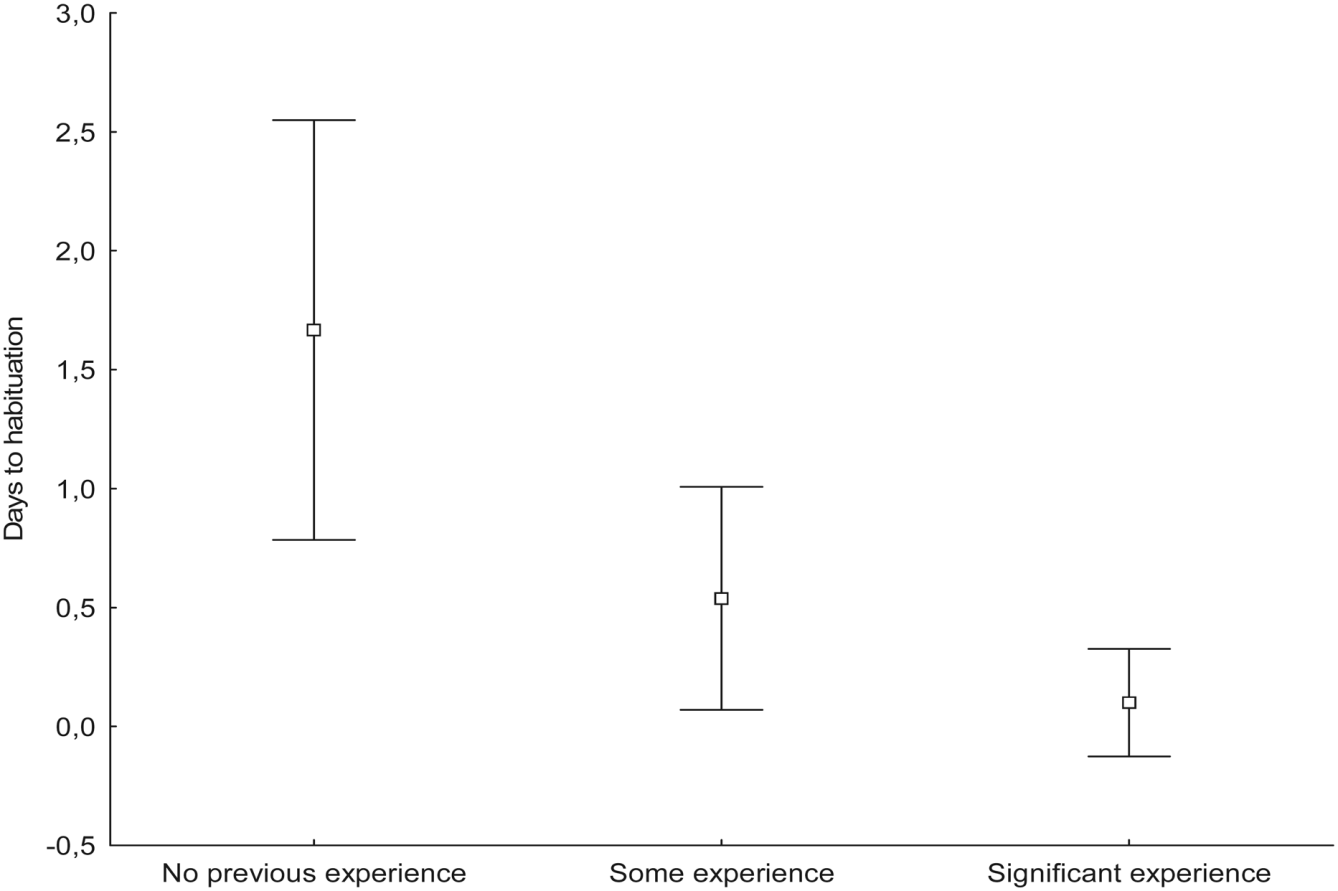
Influence of previous experience of breeding Goshawks on habituation to camera presence. *Some experience* refers to females of known identity that had a trail-camera in their nest for the second time. Females with *significant experience* had a camera in their nest for the third or fourth time. Error bars indicate 95% confidence interval of the mean.

Habituation to the camera was defined as the number of days needed for the daily number of visits to the nest and numbers of prey provided to stabilize after camera installation. Based on this definition, 58% of breeding pairs habituated to camera presence the day after camera installation; 25%, two days after installation; and 17%, more than two days after. The number of days to habituation was significantly lower among breeding pairs that had previous experience with cameras than among those that had no experience (Krukal—Wallis test, *p* = 0.023; Fig 1).

## Discussion

This study shows that trail-cameras can be a powerful method for studying the diet of nesting raptors, and can perform better than indirect methods because in principle they can detect every prey delivered to nests. However, the technique is limited by technical failures and difficulties in identifying certain prey types. This argues for using both remote direct methods and conventional indirect methods to make a more comprehensive assessment of diet. Our study also shows that adult Goshawks can alter their behavior in camera presence, an aspect that must be controlled to minimize potential negative impacts.

### Comparison of diet estimates obtained from trail-cameras and indirect methods

Camera images detected many more prey items than any of the four indirect methods. Our cameras allowed continuous observation of the nests, providing extremely high sampling intensity and thereby registering nearly every prey delivery. This leads to better estimates of prey provisioning rates and may reduce the risk of biases in diet assessment. On the contrary, estimates from prey remains may suffer from bias from the start. Many preys can be plucked outside the nesting area, and can be delivered directly to the nest leaving few remains in the nesting site to be collected [8]. In general, this might be the main reason why feather-remains from many prey deliveries are never found in typical field studies. On the other side, prey remains collected may not only represent the preys delivered to the juveniles, but also the preys eaten by the adults themselves. As these preys are typically eaten away from the nest, they are not observed in camera images, but their remains can actually be found.

Despite the high sampling intensity of the cameras, our direct method still shows potential for bias, since it did not allow us to identify all prey to species level. While we were able to identify most prey remains (bones, feathers and hair) with success similar to that of other authors [35, 36], we were unable to identify 32% of prey items in camera images to species level. In fact, 20% could not be identified to class level as bird, mammal or reptile. These percentages are similar to or lower than those reported in video-based studies of Goshawk diet [16, 18, 37]. This may give rise to bias in camera-based diet estimates. Nevertheless, we believe that this is not an important issue in our study, since we succeeded in characterizing the bias by analyzing the size of unidentified prey.

Many factors complicated prey identification to species level from camera images (Table 5). We found it difficult to differentiate bird species with similar plumage and to detect very small birds (< 100 g) in camera images; feather analysis was the most efficient method for detecting these species. Goshawks usually pluck small birds to a lesser extent than they pluck large birds at the capture site [8]; as a result, small-bird remains are abundant around the nests. Analysis of feather remains and of pellets proved valuable for compensating for deficiencies in camera analysis within the bird and mammal taxonomic classes. Camera analysis also proved inefficient at detecting small mammals (*e*.*g*. rats and micromammals), as did all indirect methods except pellet analysis. Small mammals are often consumed completely and quickly, generating few or no uneaten remains and showing up on few camera images. However, they give rise to abundant hair inside pellets. These findings argue for using both remote direct methods and conventional indirect methods to improve prey identification to species level for some prey types.

**Table 5:** Major difficulties encountered when identifying prey to species level from camera images.

Few pictures of small prey due to short handling time in the nest (see also [19])
Prey too mutilated or plucked to allow identification based on morphological features (see also [38])
Inability to distinguish between species with similar plumage (e.g. pigeons and doves)
Prey hidden behind nestlings or adults during handling
Pictures too dark or too bright due to changes in lighting related to weather or time of day (see also [23])
Low image quality that prevented identification of prey features even after enlarging the image

Camera-recording provided probably the most reliable estimates of the proportions of birds, mammals and reptiles in the diet. It detected a greater proportion of mammals than indirect methods (except pellets), and it was the only technique that detected reptiles. While feathers allow differentiating individuals within bird species by looking for the repetition of principal flight feathers or comparing the degree of development of the feathers, hair remains do not. As a result, feather-and-hair remains analysis underestimated the proportion of mammals and hence overestimated the proportion of birds. Analysis of bone remains did not compensate for this bias. On the contrary, analysis of bones provided the most biased prey information in our study, greatly overestimating the proportion of medium-sized birds (200–400 g). A high proportion of the bones collected were humerus, scapulas and coracoids of pigeons and doves that often appeared joined to one another and to the sternum. This may reflect a size bias: large bones and bone groupings are more easily found than small ones, which can easily become buried in the nest, are more difficult to find on the ground and are more digestible and decomposable. As a result, the aggregate measure of feathers-fur-and-bones, often used in raptor diet studies, did not improve these estimates. This is consistent with the observation by several other authors that prey remains overestimate the proportion of birds relative to mammals [6, 7, 38–40]. Among the indirect methods we tested, estimating the proportions of feathers and hairs in pellets produced the most similar prey estimates of birds and mammals to camera analysis, although it failed to detect any reptiles. In our study, nestling Goshawks rarely consumed the reptiles delivered to the nest, which would explain their absence in the pellets. At the same time, the long stay of unconsumed reptiles in the nest facilitated their detection with the cameras. Thus, camera-based methods also provided more complete information to analyze prey processing upon delivery to the nest and the degree of prey consumption.

Taken together, our data indicate that none of the 5 prey analysis methods in this study yielded completely unbiased estimates for all prey types and sizes. The sensitivity of each method depended principally on two key prey features: (1) the taxonomic group (bird, mammal or reptile), since this determines the type of remains observable (feathers, hairs, scales, bones) and their detectability in the field; and (2) the size, which influences the handling time of the prey in the nest and therefore the number of images of each prey registered, as well as the degree of prey consumption and therefore the abundance and detectability of uneaten remains. Trail—camera monitoring detected the greatest number of prey items, showed a good rate of prey identification to species level (although not the highest rate) and probably provided the most reliable estimates of diet composition of any of the 5 methods on its own. Indirect methods can complement camera images well because they are better at detecting small prey, especially analysis of pellets and feather remains; indirect methods are also better at identifying prey to species level, particularly analysis of feather remains.

### Trail-camera performance

The trail—cameras functioned satisfactorily. The three cameras installed in 2007 served as a pilot study to optimize the system that would be installed in subsequent years; even so, annual readjustments were necessary in order to improve performance or correct problems that arose. The factor that most limited camera performance was power supply. Adding new external lead—acid batteries and using high-capacity memory cards (2009 and 2011) increased the percentage of cameras that functioned well from installation to fledging from 44% to 68%. These cameras had a median operating time of approximately 60 days. Thus, the operating time of our trail—cameras was longer than that reported in other video- or photo—camera studies [16, 18, 21, 22]. We did not obtain such good results when we used external recharged batteries in 2010 instead of new batteries, perhaps because our recharging protocol or equipment was inadequate.

The considerable autonomy of our camera devices avoided the need for additional visits to the nests after installation in order to replace batteries or download data, minimizing research disturbances during the breeding season. The autonomy of our camera system also avoided the need to install device components at ground level in the densely populated study area, reducing the risk of theft or vandalism.

Cameras were programmed to take one picture per minute after being triggered by motion in the nest. While this frequency is substantially lower than the 1–2 frames per second used in other time-lapse systems, it still allowed reliable detection of prey deliveries and generated substantially less data, avoiding the need for frequent visits to nests to empty the memory card and making image analysis much faster than with conventional time—lapse systems [16, 41]. The 1-minute interval was still much shorter than the prey handling time of 1.21–5.27 g / min for Goshawks [42], ensuring that even small prey could be captured on several images.

Our trail-camera system provides several advantages as a remote monitoring system. It is easy to set up, adaptable to different nest structures and cost-effective. In addition to providing detailed data on diet, it also provides valuable information on the identity of pre-banded breeding individuals, data on diurnal and nocturnal behavior of nestlings and adults, data on nestling survival and age at fledging, and causes of nestling death. At the same time, our camera system presents some disadvantages. One is that after installation, cameras cannot usually be checked until the end of the breeding season to avoid disturbance and premature nest abandonment by the nestlings. Thus, camera malfunctions cannot be detected or corrected, leading to partial or complete loss of data from the affected devices [23, 43]. We note that the trail camera performance results obtained in the present study are certainly influenced by the specific trail camera models and configuration set up used. Trail-cameras with higher-quality lenses and light meters, greater resolution, and higher-capacity power supplies and memory cards are currently commercially available and affordable. Such equipment might have mitigated some of the problems that we faced and probably would have improved the rate of camera-based prey identification.

### Impact of trail-camera monitoring on nesting Goshawks

In the present study involving 80 nests monitored by trail—cameras, we did not observe any nest abandonment by adults in response to the presence of the cameras. High tolerance to other type of cameras installed in the nests has also been reported in previous studies of Goshawks [16, 18, 21] and other raptors [24, 44, 45]. Our findings are consistent with the suggestion that disturbances at nesting sites rarely cause parental desertion if they are only moderately invasive and if they occur when nestlings are not too young [46].

The breeding adults in our study exhibited some distrust towards the cameras immediately after installation, which led to a delay of several hours in the resumption of some activities such as nest cleaning (removal of prey remains, supply of green material) and prey provisioning. We also observed a delay of 1–2 days until adults became completely habituated to the presence of the cameras. We found that this delay to become completely habituated varied inversely with how much experience the adults had had with cameras. Providing supplementary food to the nest on the day of camera installation may reduce the potential effects of the temporary absence of adults due to research disturbance. In our study, the nestlings usually fed themselves on the food that we left in the nest just after camera installation, and adult females also used this extra food to feed their offspring.

Contrary to our expectations, painting the trail—cameras with camouflage colors did not shorten the time needed for nest activities to normalize. This likely reflects the relatively large size of our cameras (H × W × D = 28 × 19 × 10 cm) and their placement quite near the nest in a conspicuous, vertical position. As a result, simply painting them was not enough for them to go unnoticed. Several other authors [16, 18, 44] installed smaller cameras in the nests (*e*.*g*. H × W = 12 × 3.5 cm, [18]), and they installed the other elements of the recording system, such as the data recorder and power supply, on the ground. They did not report significant disturbance to raptor behavior, though it is unclear whether they analyzed this possibility in detail. We suspect that disturbance of adult behavior in our study would have been considerably less if we had used smaller trail—cameras currently available on the market. To minimize potential negative effects of cameras on breeding raptors we recommend using cameras that are as small as possible and installing them as quickly as possible when nestlings are not too young. Visits to the nest for camera maintenance should be avoided and pilot studies should be conducted if the researchers are unsure of the sensitivity of the raptor species to camera monitoring.

## Supporting Information

S1 Appendix Supplementary information on the trail-camera device installed in Goshawk nests. (PDF)

S1 Dataset Breeding Goshawk diet estimates. Percentage of individuals of each prey category per territory (n = 20) obtained by direct camera monitoring and four indirect analyses of prey remains collected from the nests and surroundings (pellets, bones, feather-and-hair remains, and feather-hair-and-bone remains combined).
(XLSX)

S2 Dataset Breeding Goshawk activity after camera installation. Date and time of camera installation, first observed activity of adults in the nest and first observed prey delivery. *Habituation date* is the date when the daily number of visits to the nest and number of prey provided had stabilized after camera installation. *Experience with camera* is the number of times that a breeding female had a camera in its nest.
(XLSX)

S3 Dataset Trail-camera performance. Characteristics, operating times and frequencies and types of technical failures of the trail-cameras.
(XLSX)
