# Supplementary material for: Evaluation of Trail-Cameras for Analyzing the Diet of Nesting Raptors Using the Northern Goshawk as a Model
Source: PLoS One. 2015 May 20;10(5):e0127585. doi: 10.1371/journal.pone.0127585 (PMC4438871; doi:10.1371/journal.pone.0127585)
Supplement: S1 Appendix — (PDF) [file pone.0127585.s001.pdf]

S1 Appendix. Supplementary information on the trail-camera device installed in Goshawk nests.

Evaluation of trail-cameras for analyzing the diet of nesting raptors using the Northern Goshawk as a model. Plos One. G. García-Salgado*, S. Rebollo, L. Pérez-Camacho, S. Martínez-Hesterkamp, A. Navarro and J.M. Fernández-Pereira

Ecology and Forest Restoration Group, Department of Life Sciences, University of Alcalá, Sciences Building, 28805 Alcalá de Henares, Madrid, Spain.e-mail: *gonzalo.garcias@uah.es

Two commercially available, waterproof trail-cameras were used in our study: the *Bushnell Trailscout 2.1MP* and the *Moultrie Game Spy I40*. Both cameras have approximate dimensions of H × W × D = 28 × 19 × 10 cm. Both models have a fixed focus and integrate the camera, recorder, motion sensor and energy supply into a single device. The motion sensor is a passive infrared-triggered system (Swann *et al*. 2011). Whenever possible, the cameras were installed on the branches supporting the nest platform, at a distance of 1.3 – 2 m from its center and slightly above it, in order to obtain a complete view of the nest. The cameras were mounted on adjustable metal camera brackets that comprised 2 ball-and-socket joints and that were attached to the tree branch with screws. From 2009 to 2011, the brackets were reinforced with thread-locking glue and the cameras were fixed to the tree using adjustable straps to prevent accidental movement and misframing. The cameras were focused using a built-in laser pointer and a handheld 2-inch picture viewer (Moultrie). The Bushnell and Moultrie cameras contained different built-in power supply sources. From 2008 to 2011, we connected additional external batteries to lengthen the operating time of the cameras. The specific type of external batteries used affected camera performance. The external batteries were housed in a waterproof electrical junction box measuring approximately 16 × 14 × 10 cm that was attached with duct tape to the branch behind the camera. Cameras were programmed to take one picture per minute after being triggered by motion in the nest; otherwise, the cameras did not take pictures. The time between camera activation and image capture was slightly longer than 1 second in both cameras. The incandescent flash of the Bushnell camera used in 2007 was deactivated, and these cameras were scheduled to take pictures only during daylight hours (7.00 *am* – 11.00

pm). Moultrie cameras were equipped with infrared LED flashes that we attenuated by covering with a piece of white paper (80 g/m^2). Moultrie cameras remained active 24 hours a day, recording color pictures by day and black and white pictures overnight. The three Bushnell cameras were programmed to take high-resolution pictures (1600 × 1200 pixels, ~ 0.5 Mb). Three Moultrie cameras were programmed to shoot in high resolution, while the remaining 74 shot in low resolution (640 × 480 pixels, ~ 0.085 Mb) in order to reduce individual image size and maximize operating time. Regardless of file size, both camera models are limited to storing a maximum of 9999 pictures. The date, time and nesting territory code were encoded in each image. The cost of the entire system (trail-camera, bracket and batteries) did not exceed 190 € (~ 260 USD) per device.

Year	Nests	Camera brand-model	Internal batteries	Customized external batteries	Memory card
2007	3	Bushnell Trailscout 2.1Mp	4 × long-life IEC LR20 1,5V	NA	SD 1 Gb
2008	18	Moultrie Game Spy I40	6 × long-life IEC LR20 1,5V	6 × long-life IEC LR20 1,5V	SDHC 4 Gb
2009	19	Moultrie Game Spy I40	6 × IEC LR20 1,5V	2 × sealed lead acid batteries12 V 7Ah (new)	SDHC 4 Gb
2010	21	Moultrie Game Spy I40	6 × IEC LR20 1,5V	2 × sealed lead acid batteries12 V 7Ah (recharged)	SDHC 4 Gb
2011	19	Moultrie Game Spy I40	6 × IEC LR20 1,5V	2 × sealed lead acid batteries12 V 7Ah (new)	SDHC 4 Gb
